# Promoting Nordic ‘Green Care Practices’

**DOI:** 10.1080/02813432.2025.2503450

**Published:** 2025-05-16

**Authors:** Nanna Holt Jessen, Stefan Hjørleifsson

**Affiliations:** ^a^ Research Unit for General Practice, Aarhus, Denmark; ^b^ Department of Global Public Health and Primary Care, University of Bergen, Bergen, Norway

**Keywords:** General practice, sustainability, green care, public health, climate mitigation

## Abstract

Climate change poses an urgent threat to human health, with thousands of deaths already attributed to extreme heat. In the Nordic countries, where per capita emissions are particularly high, healthcare accounts for 4.2–6.3% of emissions, highlighting the need for immediate action to reduce its climate impact. Evidence supporting sustainable climate solutions in healthcare is steadily growing. Greenhouse gas emissions in general practice can be broadly divided into those arising from clinical activities, such as patient examination, diagnosis, and treatment, and those from non-clinical activities, including clinic operations, energy use, and transportation by staff and patients. In this opinion paper, we discuss and advocate ‘Green Care Practices’ to minimize greenhouse gas emissions specifically related to clinical activities. We believe that Nordic general practitioners can adopt ‘Green Care’ to significantly reduce healthcare-related emissions while improving patient outcomes, enhancing efficiency, and promoting environmentally responsible healthcare. Strong primary healthcare, rooted in Nordic values, can support the United Nations Sustainable Development Goals by simultaneously mitigating climate change, reducing inequalities, fostering community stability, and promoting health and well-being. Thus, GPs can ensure sustainability for future generations.

## KEY MESSAGES

- Climate change is an urgent threat to human health, with healthcare in the Nordic countries contributing ∼5% of national emissions, emphasizing the need for immediate action.
- ‘Green Care Practices’ can significantly reduce greenhouse gas emissions from clinical activities, improving both environmental and patient health outcomes.
- Nordic GPs can align with the UN Sustainable Development Goals, fostering health, equality, and environmental responsibility for future generations.

## Introduction

Globally, several national colleges of general practitioners (GPs) have started to address the climate crisis [1–3]. Research indicates that GPs are concerned about sustainable development and are actively engaged in its implementation in their practice [4–6]. As healthcare professionals, GPs strive to diagnose and treat without causing harm to the individual or to society [7,8]. Given the ongoing climate crisis, this awareness now includes consideration to minimize harm to the environment [6,9]. Strong primary healthcare, as outlined in the Core Values and Principles of Nordic General Practice/Family Medicine [7,10], can be an essential cornerstone of sustainable and socially responsible healthcare, aligning and promoting the United Nations Sustainable Development Goals (SDGs). For example, locally delivered and universally available primary healthcare contributes to reduced inequalities (SDG 10), stable communities (SDG 11), and responsible production and consumption (SDG 12), in addition to good health and well-being (SDG 3). By further incorporating protection of the environment and the climate in their activities, GPs might be able to ensure that general practice in the Nordic countries become ‘Green Care Practices’ to reduce the extent of climate crisis (SDG 13) and increase the likelihood that the needs of future generations can be met.

In this opinion paper, we discuss initiatives to reduce greenhouse gas emission from general practice and more generally to promote ‘Green Care Practices’. Our point of departure includes the conviction that general practice in the Nordic countries is well situated and has a moral obligation to provide ‘Green Care’. NHJ is involved in the ‘Green Practice’ initiative of the Danish College of General Practitioners and has co-authored the Danish college’s new sustainability policy [3]. SH is the chair of Choosing Wisely Norway and leads the ‘Sustainability in general practice’ project of the Norwegian College of General Practitioners [11].

## Climate change, health, and healthcare

As acknowledged by the WHO, ‘Climate change presents a fundamental threat to human health’ [12]. This is not just a future, hypothetical threat. It has been estimated that during the summer of 2022 heat caused more than 60,000 deaths in Europe, and that more than 47,000 people died in the region as a result of high temperatures in 2023 [13,14]. Globally, climate change is expected to cause ∼250,000 additional deaths per year between 2030 and 2050 [12].

Whereas awareness of the health consequences of climate change and the need for capacity building and climate resilience in health systems is not new, the urgent need to reduce climate emissions from health systems has become more of a focus in recent years. Globally, it is estimated that the healthcare sector is responsible for 4.4% of the total carbon footprint [15]. Keeping in mind that the per capita carbon emissions in the Nordic countries are extremely high, we must acknowledge that the healthcare sector proportion of the total national carbon emissions is 4.2% in Norway, 4.4% in Sweden, and an astonishing 6.3% in Denmark [15].

## National and international policies

The UK National Health Service was the first to pledge to reduce greenhouse gas emissions from healthcare as early as in 2008. They identified ‘carbon hotspots’ within healthcare, successfully lowering emissions while treating more patients. A significant portion of emissions from general practice was found to stem from the use of medications.

In 2019, the Royal College of General Practitioners in the UK recognized climate change as a health crisis, urging GPs to act. They developed the Green Impact for Healthcare Toolkit to promote sustainability in general practice. Similarly, Ireland’s Irish College of General Practitioners has developed a toolkit with a broad range of recommendations, from appropriate prescribing of medications and disposal of inhalers to non-medical prescriptions.

Human health was brought to the forefront of climate change work at the 26th United Nations Climate Change Conference (COP26) in 2021. Eighty countries have joined the UN Alliance for Transformative Action on Climate and Health that was launched at COP26 to not only strengthen climate resilience but also to lower the emissions of greenhouse gases from their national health systems. However, among the Nordic countries, only Norway has committed to these goals.

In Denmark, the Danish Medical Association published a climate policy in 2022, stating that climate policy is health policy, and advocating for public health to be a key component of the national climate strategy [16]. In 2024, the Danish College of General Practitioners released a sustainability policy titled ‘From Globe to Clinic’ (‘Fra Klode til klinik’) outlining how sustainability can be and already partly is integrated into Danish general practice across social, economic, environmental, and climate dimensions [3].

## Current knowledge and practices in general practice

There is increasing evidence in support of sustainable climate solutions in healthcare. Greenhouse gas emissions in general practice can be categorized according to whether they stem from clinical activities, which include the examination, diagnosis, and treatment of patients, or non-clinical activities, which can involve clinic operations, electricity, heating, and transportation for staff and patients. In the following, we will focus on how future ‘Green Care Practices’ can reduce the greenhouse gas emissions from clinical activities.

### Medical prescriptions

General practice accounts for 88% of medical prescriptions in Denmark, both through initiating treatment and maintaining those started at hospitals or by private specialists [17]. Data from England estimates that 48% of general practice’s greenhouse gas emissions come from prescribing medications alone [18].

The United Nations estimates that phasing out hydrofluorocarbon (HFC) gases could reduce global warming by 0.4 °C by the end of the century, with significant implications for future health and patient safety [19]. Among other uses, such gases are important propellants in spray inhalers used for asthma and chronic obstructive pulmonary disease (COPD). The two most used HFC gasses in spray inhalers are 1300 and 3350 times more harmful to the climate than CO_2_, respectively, and spray inhalers thus have a 50–100 times higher climate impact over a 20-year period than equivalent powder inhalers [20].

Since 2022, the Danish Health Authority has recommended powder inhalers over spray inhalers to treat asthma and COPD where possible [21]. In Norway, the climate poster for general practice encourages GPs to use powder rather than spray inhalers, and GPs have also received reminders and information about this topic from the College and from the Directorate of health [11].

Long-term prescriptions and polypharmacy entail waste, harm to patients, and large amounts of emissions. Between 30 and 50% of medicines prescribed for long-term conditions are not taken as intended, and in a recent study in Norway, one out of five visits to a hospital emergency department were related to medication use [22]. The harms of polypharmacy include confusion, falls, weakness, loss of appetite, and gastrointestinal problems. Across Europe, one out of five adults in the age group 70–74 take ten or more medications per day, and in England a national review estimated that 10% of all medicines prescribed in primary care are overprescribed [23,24]. Evidence suggests, however, that most patients with polypharmacy will accept deprescribing if their doctor says it is safe [25]. Environmentally friendly treatment options should also be chosen in the first place rather than medications. Inspiration can be found in a systematic review from 2024 indicating that physical activity is effective for treating depression, with effects comparable to psychotherapy and pharmacotherapy [26].

Besides greenhouse gas outputs, excessive use of antibiotics in healthcare and especially in agriculture contributes significantly to antimicrobial resistance (AMR), posing a substantial risk to patient safety. Among European countries, Denmark ranks highest in antibiotic prescriptions in nursing homes. However, simple strategies, such as improving communication between nursing home staff and GPs, have been shown to nearly halve antibiotic prescriptions for urinary tract infections without increasing the incidence of hospitalization [27].

### Surgical equipment

The use of medical instruments in general practice also leads to emissions of greenhouse gases. A comparison between reusable steel and disposable acrylic vaginal specula for gynecological examinations shows significant CO_2_ differences, with reusable steel specula having a lower overall climate footprint [28]. Although disposable specula have a smaller CO_2_e footprint per item, the need for a new speculum for each examination increases the total footprint. Studies confirm that autoclave sterilization is highly effective and that single-use equipment used in surgical settings is generally more harmful than equivalent reusable items across a range of environmental parameters [29].

### Non-medical prescriptions

‘Social prescribing’ was originally endorsed by the NHS to allow healthcare professionals, including GPs to refer patients to a ‘link worker’ [30] who collaborates with the patient to identify locally community- and nature-based activities that can promote social, mental, and physical health, often through physical activity [31,32]. Additionally, research indicates that exposure to nature may foster a stronger sense of ‘nature connectedness’, which in turn encourages more environmentally friendly and sustainable behaviors [33,34]. A recent systematic review suggests that social prescribing may also reduce the frequency of GP and hospital visits, potentially decreasing the greenhouse gas emissions of clinical activities [35,36].

### Overdiagnosis and overtreatment

Research indicates that physician-ordered tests and treatments fall into the categories of ‘good value care’ in 60% of cases, ‘low value care’ in 30%, and ‘harmful care’ in the remaining 10% [37,38]. It is urgent to identify the 40% of tests and treatments that do not provide meaningful benefits for patients’ health or quality of life and may cause harm [39]. With patient safety as a central priority, efforts must focus on reducing both harmful and low-value care practices, which will concurrently help the healthcare sector reduce its greenhouse gas emissions.

The ‘Choosing Wisely’ initiative to reduce overdiagnosis and overtreatment has gained foothold in Denmark, Finland, Sweden, Norway, and 31 other countries [40,41]. In Denmark, the Choosing Wisely initiative ‘Vælg klogt’ is a collaborative effort between the national patient organization and the Danish Medical Societies and is thus the first of its kind with patients and doctors jointly leading the organization as equal partners. The Norwegian Choosing Wisely initiative ‘Gjør kloke valg’ that was established by the Norwegian medical association in 2018, is distinguished by a close collaboration with the national colleges of physiotherapists, psychotherapists, nurses, dentists, manual therapists, ophthalmologists, radiotherapists, chiropractors, pharmacists, biomedical laboratory scientists and midwifes. Choosing Wisely marks an essential cultural shift to avoid low-value investigations and treatments, and decreasing greenhouse gas emissions is an important co-benefit, without risks or downsides for patients. Fortunately, much is already known about the many drivers of medical overuse and how to counter them [42].

### Tests

GP initiated overuse of blood tests, radiological imaging and other tests can lead to overdiagnosis and cascades of investigations and overtreatment that sometimes extend into hospital services [43]. While it is increasingly recognized that such overuse entails waste of healthcare resources and sometimes causes harm to patients, the climate implications of over-testing must also be acknowledged. Each test has a climate impact, and each case of over-investigating can initiate management cascades with additional emissions of greenhouse gases. Avoiding overuse of tests is therefore crucial to reducing greenhouse gas emissions. Eliminating unnecessary tests from the shortcut menu of laboratory test ordering systems is a promising approach to reduce overuse of tests [44].

An example is D-vitamin testing. Around 20 years ago, the potential importance of D-vitamin deficiency for various diseases gained attention, leading to a rise in D-vitamin testing. While adequate D-vitamin intake is essential to avoid rickets and osteomalacia, Norwegian and Danish healthcare authorities recommend dietary intake or use of D-vitamin supplements without the need for blood tests or prescriptions [45,46]. An Australian study found that testing was of low value in around 75% of cases and estimated a reduction of 28,576 kg CO_2_e annually by decreasing unnecessary D-vitamin tests [47].

## Conclusion

GPs in the Nordic countries have the potential to implement ‘Green Care’ initiatives, which can lead to a significant reduction in greenhouse gas emissions associated with healthcare delivery. By transitioning toward ‘Green Care’, these efforts not only contribute to mitigating climate change but also yield important co-benefits for both patients and the broader healthcare system. Such benefits may include improved health outcomes through sustainable practices, enhanced operational efficiency, and fostering a more environmentally responsible healthcare model.

High-quality, robust primary healthcare, deeply rooted in Nordic values, plays a vital role in advancing the SDGs. By reducing inequalities, fostering stable and resilient communities, and ensuring good health and well-being for all, it serves as a foundation for socially responsible and sustainable healthcare. We believe that the time has come to extend the sustainability perspective of general practice to also include caring for the climate and our planet. By adopting ‘Green Care Practices’, GPs can contribute to the mitigation of climate change and safeguarding the health and well-being of future generations, ensuring that healthcare systems remain effective and sustainable.
